# Inhibition of the angiotensin-converting enzyme N-terminal catalytic domain prevents endogenous opioid degradation in brain tissue

**DOI:** 10.1172/jci.insight.194624

**Published:** 2025-11-18

**Authors:** Filip Hanak, Jessica L. Swanson, Krzysztof Felczak, Prakashkumar Dobariya, Ursula C.H. Girdwood, Kenneth E. Bernstein, Swati S. More, Patrick E. Rothwell

**Affiliations:** 1 Department of Neuroscience, University of Minnesota Medical School, Minneapolis, Minnesota, USA.; 2 Center for Drug Design, College of Pharmacy, University of Minnesota, Minneapolis, Minnesota, USA.; 3 Department of Pathology and Laboratory Medicine, Cedars-Sinai Medical Center, Los Angeles, California, USA.

**Keywords:** Neuroscience, Vascular biology, Peptides, Psychiatric diseases

## Abstract

Natural opioid signals in the brain produced by Met-enkephalin-Arg-Phe are enhanced after blocking its degradation by the N-terminal catalytic domain of angiotensin-converting enzyme.

**To the Editor:** Angiotensin-converting enzyme (ACE) is a dipeptidyl carboxypeptidase, cleaving 2 amino acids from the C-terminus of peptide substrates (1). ACE is well known for regulating blood pressure by converting angiotensin I to angiotensin II. We recently identified another role for ACE in the brain (2): modulating synaptic plasticity by cleaving and degrading Met-enkephalin-Arg-Phe (MERF), an endogenous opioid peptide. ACE inhibition prevented MERF degradation (Supplemental Figure 1; supplemental material available online with this article; https://doi.org/10.1172/jci.insight.194624DS1) and enhanced endogenous opioid signaling in the nucleus accumbens, a brain region with conjointly high expression of ACE, MERF, and cognate opioid receptors. This could plausibly explain clinical reports that centrally active ACE inhibitors have unexpected secondary benefits (see Supplemental Text). However, the mechanism by which ACE cleaves MERF and regulates endogenous opioid signaling in brain tissue remains poorly understood.

ACE has 2 catalytic domains, located in the N-terminal or C-terminal region of the protein (Figure 1A), with distinct profiles of substrate specificity (1). To investigate the contribution of each catalytic domain to MERF degradation, we used liquid chromatography–tandem mass spectrometry (LC-MS/MS) to quantify extracellular enkephalin in mouse brain tissue (2, 3). Acute coronal brain slices containing the nucleus accumbens were incubated in artificial cerebrospinal fluid containing a saturating concentration of exogenous MERF, which is cleaved and degraded by ACE to produce Met-enkephalin (Figure 1B). To study the role of each catalytic domain in MERF degradation, we used mouse lines carrying mutations in the active site of either the N-terminal catalytic domain (NKO) or the C-terminal catalytic domain (CKO; Figure 1C). Crucially, these mutations are amino acid substitutions that do not change the expression level of ACE and preserve the function of the intact domain (4, 5). The NKO mutation significantly reduced conversion of exogenous MERF to Met-enkephalin (Figure 1D), whereas the CKO mutation had no effect (Figure 1E). We complemented this genetic analysis with acute pharmacological inhibition using RXP407 and RXPA380, small molecule ACE inhibitors with a high selectivity for the N-terminal and C-terminal catalytic domains, respectively (Figure 1F). RXP407 reduced Met-enkephalin production in a dose-dependent manner (Figure 1G), recapitulating our findings with NKO mice, whereas RXPA380 had no effect (Figure 1H).

To build on our analysis of exogenous MERF degradation by ACE, we next measured the degradation of endogenous MERF released from brain tissue following chemical stimulation with a high concentration of potassium chloride (Figure 1I). In the presence of RXP407, we observed a dose-dependent increase in MERF concentration (Figure 1J), but no change in the concentration of endogenous Met-enkephalin or Leu-enkephalin (Supplemental Figure 2, A and B). RXP407 had no effect in NKO mice (Supplemental Figure 2C), ruling out nonspecific effects of RXP407 on other targets. To measure the functional impact of RXP407 on synaptic transmission, we prepared acute brain slices from Drd1-tdTomato reporter mice, to guide whole-cell patch-clamp recordings from individual Drd1-expressing medium spiny neurons in the nucleus accumbens (Figure 1K and Supplemental Figure 3). Bath application of RXP407 caused long-term depression of electrically evoked excitatory postsynaptic currents (Figure 1L), as well as an increase in the paired-pulse ratio (Figure 1M), both consistent with our prior report that elevated MERF levels reduce presynaptic glutamate release (2). Please see Supplemental Methods for a methodology description.

Our results suggest that the ACE N-terminal catalytic domain is the primary site of MERF degradation in brain tissue, and that N-terminal domain inhibition is sufficient to reduce degradation of this specific endogenous opioid peptide. This conclusion is further supported by structural modeling of the probable conformation of MERF in the ACE N-terminal catalytic domain (Supplemental Figure 4), with productive catalysis due to efficient transition state stabilization, as well as conserved active site interactions previously established for domain selectivity (6). Pharmacological inhibition of the ACE N-terminal catalytic domain thus presents what we believe to be a novel strategy to enhance endogenous opioid signaling in the brain. Critically, we have previously shown that central ACE inhibition does not have obvious rewarding effects, and in fact attenuates the rewarding properties of exogenous opioids like fentanyl (2). The engagement of endogenous opioid signaling without corresponding risk of misuse would be a major advance for opioid-based pharmacotherapy, with translational potential for various neuropsychiatric conditions (see Supplemental Text). Our current work will guide the development of more specific ACE inhibitors that target the primary site of MERF degradation in the brain, resulting in tailored pharmacotherapies that target the endogenous opioid system with better efficacy and fewer side effects.

## Funding support

This work is the result of NIH funding, in whole or in part, and is subject to the NIH Public Access Policy. Through acceptance of this federal funding, the NIH has been given a right to make the work publicly available in PubMed Central.

- NIH grants DA060664 (to JLS), DA056331 (to SSM and PER), and DA056675 (to SSM and PER).
- University of Minnesota’s MnDRIVE (Minnesota’s Discovery, Research, and Innovation Economy) initiative (to FH and PER).
- University of Minnesota Graduate School Interdisciplinary Doctoral Fellowship (to FH).
- University of Minnesota Office of Academic Clinical Affairs Faculty Research Development Grant (to SSM and PER).
- NIH/National Cancer Institute Cancer Center Support Grant CA-77598 to the Analytical Biochemistry Shared Resource of the Masonic Cancer Center (support for mass spectrometry).

## Supplementary Material

Supplemental data

Supporting data values

## Figures and Tables

**Figure 1:**
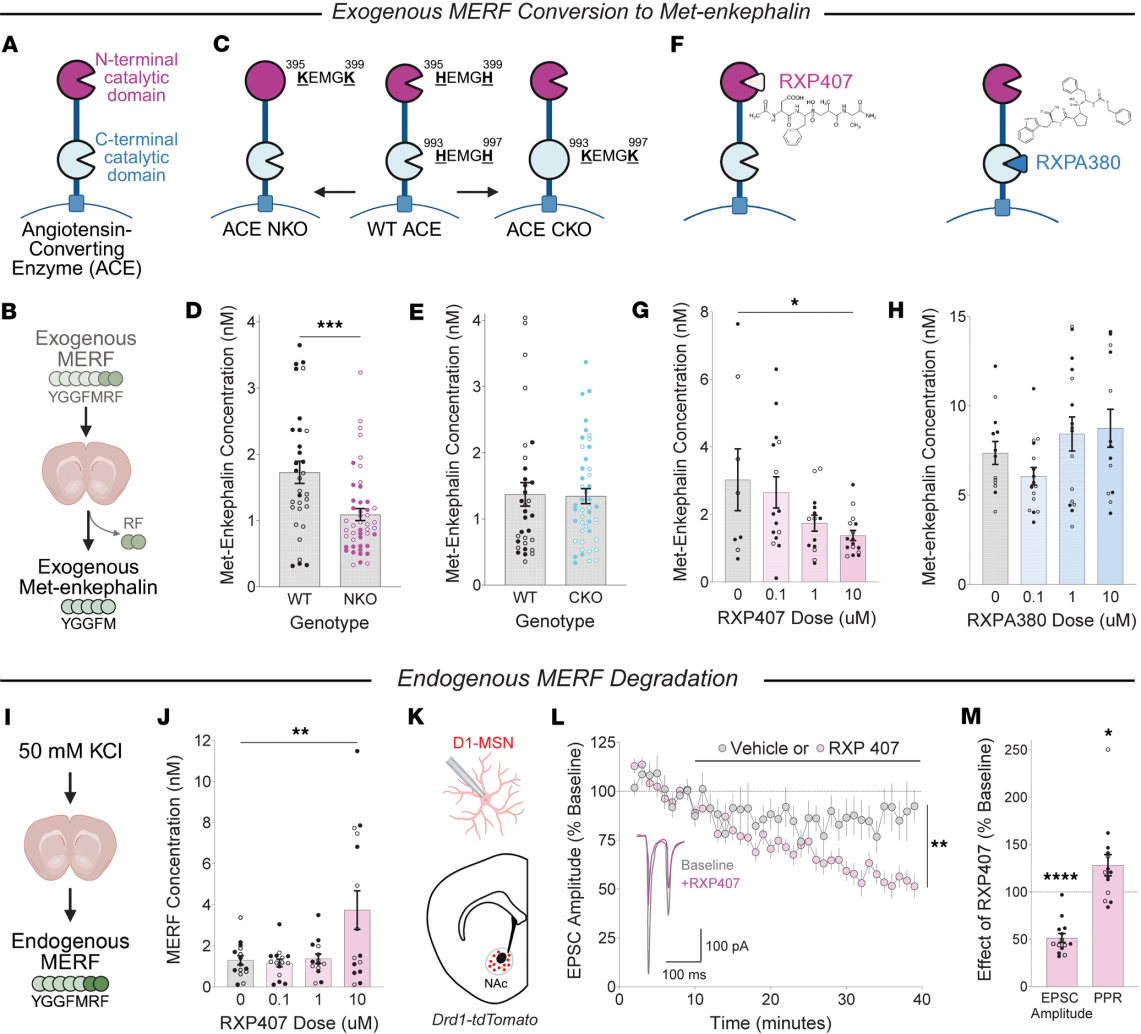
Met-enkephalin-Arg-Phe (MERF) degradation by the angiotensin-converting enzyme (ACE) N-terminal catalytic domain. (**A**) Illustration of ACE catalytic domains. (**B**) Experimental design: Exogenous MERF was applied to brain slices and cleaved by ACE to produce Met-enkephalin. (**C**) Genetic mutations inactivating the ACE N-terminal domain (NKO) or C-terminal domain (CKO). (**D**) MERF conversion to Met-enkephalin in NKO mice (*n* = 47) and wild-type (WT) littermates (*n* = 32). ***P < 0.001 by 1-way ANOVA, genotype main effect. (**E**) MERF conversion to Met-enkephalin in CKO mice (*n* = 45) and WT littermates (*n* = 32). (**F**) Pharmacological inhibition of ACE N- and C-terminal domains with RXP407 and RXPA380, respectively. (**G**) Effects of RXP407 on MERF conversion to Met-enkephalin (*n* = 8–16/dose). *P < 0.05 by Dunnett’s post hoc test after significant 1-way ANOVA dose main effect (*P* < 0.05). (**H**) Effects of RXPA380 on MERF conversion to Met-enkephalin (*n* = 13–16/dose). (**I**) Experimental design: Stimulation of brain slices with 50 mM KCl to release endogenous MERF. (**J**) Effects of RXP407 on MERF extracellular concentration (*n* = 15–16/dose). **P < 0.005 by Dunnett’s post hoc test after significant 1-way ANOVA dose main effect (*P* < 0.01). (**K**) Whole-cell patch-clamp recording from nucleus accumbens Drd1-expressing medium spiny neuron (D1-MSN). (**L**) Bath application of RXP407 (10 μM, *n* = 15) caused long-term depression of evoked excitatory postsynaptic current (EPSC) amplitude, which was significantly greater than vehicle (*n* = 10). **P < 0.01 by 2-way ANOVA, treatment × time interaction. (**M**) Effects of RXP407 on EPSC amplitude and paired-pulse ratio. *P < 0.05, ****P < 0.0001 by 1-sample *t* test versus reference value (100%). Graphs display mean ± SEM, with individual data points from female and male mice shown as open and closed circles, respectively.

## Version 1 11/18/2025

In-Press Preview

## Version 2 01/09/2026

Electronic publication
